# Deletion of NEMO Inhibits EMT and Reduces Metastasis in KPC Mice

**DOI:** 10.3390/cancers13184541

**Published:** 2021-09-10

**Authors:** Miltiadis Tsesmelis, Kanishka Tiwary, Katja Steiger, Nadine Sperb, Melanie Gerstenlauer, Uta Manfras, Harald J. Maier, Patrick C. Hermann, Lap Kwan Chan, Thomas Wirth

**Affiliations:** 1 Institute of Physiological Chemistry, University of Ulm, 89081 Ulm, Germany; miltiadis.tsesmelis@uni-ulm.de (M.T.); nadine.sperb@uni-ulm.de (N.S.); melanie.gerstenlauer@uni-ulm.de (M.G.); uta.manfras@uni-ulm.de (U.M.); harald.maier@novartis.com (H.J.M.); 2 Department of Internal Medicine I, University of Ulm, 89081 Ulm, Germany; kanishka.tiwary@uni-ulm.de (K.T.); patrick.hermann@uni-ulm.de (P.C.H.); 3 Department of Pathology, School of Medicine, Technical University of Munich, 81675 Munich, Germany; katja.steiger@tum.de; 4 Novartis Pharma AG, 4056 Basel, Switzerland; 5 Institute of Molecular Cancer Research, University of Zurich, 8057 Zurich, Switzerland; 6 Department of Pathology and Molecular Pathology, University Hospital of Zurich, 8091 Zurich, Switzerland

**Keywords:** PDAC, pancreatic cancer, NEMO, NF-κB, KPC, metastasis, EMT

## Abstract

**Simple Summary:**

Pancreatic ductal adenocarcinoma (PDAC) is highly metastatic and is expected to be the second leading cause of cancer-related deaths in the USA by 2030. In the current study, we investigated the role of NEMO/NF-κB signaling in the development and metastasis of PDAC by using the genetically modified KPC mouse model. In the absence of NEMO, KPC mice exhibited extended survival, which was accompanied by a strong reduction in the development of liver metastasis and ascites. Our study provides evidence for a detrimental role of the conventional NF-κB pathway in the survival of KPC mice and underlines the fact that NF-κB could be a therapeutic target against PDAC.

**Abstract:**

Pancreatic ductal adenocarcinoma (PDAC) remains a largely incurable cancer type. Its high mortality is attributed to the lack of efficient biomarkers for early detection combined with its high metastatic properties. The aim of our study was to investigate the role of NF-κB signaling in the development and metastasis of PDAC. We used the well-established KPC mouse model, and, through genetic manipulation, we deleted NF-κB essential modulator (NEMO) in the pancreata of KPC mice. Interestingly, NEMO deletion altered the differentiation status of the primary tumor but did not significantly affect its development. However, in the absence of NEMO, the median survival of the mice was prolonged by 13.5 days (16%). In addition, examination of the liver demonstrated that, whereas KPC mice occasionally developed liver macro-metastasis, NEMO deletion completely abrogated this outcome. Further analysis of the tumor revealed that the expression of epithelial–mesenchymal transition (EMT) transcription factors was diminished in the absence of NEMO. Conclusively, our study provides evidence that NF-κB is dispensable for the progression of high-grade PanINs towards PDAC. In contrast, NF-κB signaling is essential for the development of metastasis by regulating the gene expression program of EMT.

## 1. Introduction

Pancreatic ductal adenocarcinoma (PDAC) represents the most common form of pancreatic cancer and remains one of the deadliest cancer types, with an overall 5-year survival rate of approximately 9%. It is expected to be the second leading cause of cancer-related deaths in the USA by 2030 [1,2]. Its high mortality can be attributed to the lack of clear symptoms and efficient biomarkers for early detection, combined with the high invasive properties of the cancer [3,4]. Current strategies against pancreatic cancer involve treatment with chemotherapeutic agents such as gemcitabine and FOLFIRINOX [5]. However, this approach increases the survival of the patients only by a few months since in most cases, at the time of diagnosis, the tumor has already spread and metastasized [5]. Thus, it is of utmost importance to achieve a better molecular understanding of the underlying pathomechanisms in order to develop new therapeutic strategies against PDAC.

PDAC primarily arises from precancerous lesions called pancreatic intraepithelial neoplasias (PanINs) [6]. PanINs are histologically categorized into individual grades: PanIN1A, PanIN1B, PanIN2 and PanIN3. Each ensuing grade is characterized by the accumulation of additional mutations as well as by an increase in the cytonuclear and architectural atypia [6,7]. Molecular analyses have identified that low-grade PanINs (PanIN1A, PanIN1B) are defined by point mutations of the KRAS oncogene, leading to its constitutive activity. Oncogenic KRAS is the most common genetic abnormality in PDAC and appears in over 95% of all PDAC cases [8]. While oncogenic KRAS expression is essential for the development of precancerous lesions, it is accompanied by the induction of oncogene-induced senescence, which serves as a protective mechanism against the development of PDAC [9]. PanIN2 lesions are characterized by mutations in p16^INK4^. Inactivation of p16^INK4^ appears in 90% of all PDAC cases and supports the escape of the precancerous lesions from the oncogene-induced senescence, promoting the progression of PanINs to the next grade [9,10]. PanIN3 lesions, also referred to as carcinoma in situ, show p53-inactivating mutations, which appear in 50–75% of all PDAC cases. This bolsters the proliferative properties of the precancerous lesions, which finally leads to the development of PDAC [9,10].

PDAC tends to primarily metastasize to the liver and to the lung [11]. Cancer cells differentiate towards a mesenchymal phenotype, in a process known as epithelial–mesenchymal transition, or, in short, EMT [12]. During EMT, cancer cells change their gene expression program, resulting in an increased expression of mesenchymal markers and a loss of specific epithelial markers. This process boosts the invasive properties of cancer cells that escape from the primary site of the tumor, migrate through the bloodstream or the lymphatic system towards distant organs and metastasize [13]. Finally, metastasized cancer cells differentiate back to their epithelial phenotype, through a process known as mesenchymal–epithelial transition (MET) to successfully establish metastatic colonies [14].

The nuclear factor κB (NF-κB) pathway is associated with the regulation of many cellular processes, such as immune response, cell proliferation as well as survival mechanisms and has been linked with carcinogenesis in different types of cancer [15,16,17,18]. NF-κB is a dimeric transcription factor with multiple potential combinations of RelA/p65, RelB, c-Rel, NFKB1/p50, and NFKB2/p52 [19]. The prototypical NF-κB heterodimer p50:p65 mainly regulates the conventional NF-κB pathway, which is known to be active in pancreatic cancer and necessary for the development of PanINs [18]. In quiescent cells, p50:p65 is attached to its inhibitor IκBα, retained in the cytoplasm and is inactive. A number of stimuli result in the activation of the IκB kinase (IKK) complex, which consists of IKKα, IKKβ and NF-κB essential modulator (NEMO/IKKγ). The activated IKK complex consequently phosphorylates IκBα, which leads to its ubiquitination and proteasomal degradation. Thus, NF-κB can translocate to the nucleus and regulate transcription [20].

Activation of the NF-κB pathway is observed in 67% of all PDAC cases [21]. Mechanistically, constitutively active KRAS leads to the activation of the activator protein-1 (AP-1) complex. AP-1 induces the expression of IL-1α, which then activates the conventional NF-κB pathway [22]. In turn, activated NF-κB bestows proliferative and anti-apoptotic properties to neoplastic cells, supporting the development of PDAC [23]. In addition to its aforementioned properties, NF-κB can either promote or diminish the immune reaction in the pancreas depending on the context of the background [19,24]. For instance, in the context of chronic pancreatitis, NF-κB can protect the parenchymal compartment of the pancreas by limiting the constitutive inflammation and fibrosis [24]. However, in the context of the oncogenic KRAS expression, NF-κB induces the expression of pro-inflammatory cytokines and HES1, a suppressor of the anti-inflammatory response, which support the development of PDAC [25].

We previously studied the role of NF-κB in the development of PanINs and showed that blocking the conventional NF-κB pathway in a murine mutant KRAS-driven model dramatically reduced the development of PanINs [23]. In the current study, we investigated the role of NF-κB in the well-established Pdx1-Cre; KRAS^G12D^; p53^fl/fl^ (KPC) mouse model, where a combination of constitutively active KRAS and p53 deletion strongly accelerates the development of pancreatic cancer. Abrogation of the conventional IKK/NF-κB signaling by deleting *inhibitor of NF-κB kinase regulatory subunit gamma* (*Ikbkg*), which encodes NEMO, through genetic manipulation did not significantly affect the development of the primary tumor, but resulted in substantially reduced metastasis and prolonged survival of the mice.

## 2. Material and Methods

### 2.1. Mice

The mouse models were generated by crossing mice expressing Cre recombinase under the Pdx-1 promoter [26] with mice carrying an LSL-KRAS^G12D^ allele [27], floxed *p53* alleles [28], and/or a floxed *Ikbkg* allele (homozygous floxed *Ikbkg* in female) [29]. The mice (all C57BL/6) were kept at the animal facility of the University of Ulm. Littermates carrying various genotypes but not expressing Cre recombinase were used as controls and were designated as WT mice. Experiments were in accordance with German animal welfare legislation and approved by the responsible government agency.

### 2.2. RNA Isolation, cDNA Synthesis and qRT-PCR

Tissue was snap-frozen in liquid nitrogen and pulverized. mRNA was extracted from the pulverized pancreas with the RNeasy Mini Kit (Qiagen #74104). cDNA was synthesized with Transcriptor High Fidelity cDNA Synthesis Kit (Roche #5081955001). qRT-PCR was performed in Lightcycler 480 (Roche). RPL13 was used as a reference gene for relative quantification. A list of primers is shown in Table S1.

### 2.3. Protein Isolation and Western Blot

Tissue was snap-frozen in liquid nitrogen and pulverized, while cell pellet from primary cell cultures was stored at −80 °C. Pulverized pancreatic tissue or cell pellet were resuspended in buffer containing 4% sodium dodecyl sulfate (SDS), 100 mM Tris-HCl, protease and phosphatase inhibitors. Western blots were performed according to standard protocols. An antibody list is provided in the Supplementary Materials (Table S1).

### 2.4. Histology

For cryosections, tissue was snap-frozen in liquid nitrogen and preserved at −80 °C. For paraffin sections, tissue was formalin-fixed in 4% neutral buffered formalin at room temperature for 6 h, proceeded to dehydration and embedded in paraffin until sectioning. For quantitative microscopy, either the whole section, or at least 6 random fields of the section were captured with the BZ-X810 microscope (Keyence) and analyzed. Detailed hematoxylin and eosin (H&E), immunohistochemistry, immunofluorescence and Heidenhain’s azocarmine aniline blue (AZAN) staining protocols are presented in Appendix A. An antibody list is provided in the Supplementary Materials (Table S1).

### 2.5. Cancer Grading, Differentiation Status and Gross Anatomy

Cancer grading and differentiation status of the cancer were evaluated by a veterinary pathologist according to H&E-stained tissue sections. For gross anatomical analysis of the liver, pictures were captured with a conventional camera.

### 2.6. Evaluation of Ascites Development

Mice were euthanized and their peritoneal cavity was evaluated. When the peritoneal cavity was swollen and filled with ascitic fluid, the mouse was scored as positive for “ascites”. When blood was detected to the ascitic fluid, mice were scored as positive for “hemorrhagic ascites”. When no swollen peritoneum and no ascitic fluid were detected, mice were scored as “no ascites”. In some occasions, the peritoneum of the mouse was not swollen and no overt accumulation of ascites could be detected, but only a small amount of liquid in the peritoneal cavity could be observed. Mice belonging to this category were scored as positive for “slight intraabdominal exudation”.

### 2.7. Quantification of Aspartate Transaminase (AST) and Alanine Transaminase (ALT) Levels

For the quantification of AST and ALT levels, serum was isolated from the blood of the mice. A total of 30 μL of serum was placed on test strips for AST (Reflotron #10745120) or ALT (Reflotron #10745138) and the strips were then inserted to the Reflotron Plus system.

### 2.8. Cell Isolation from Ascites and Immunofluorescence Staining

Ascitic fluid was isolated from the peritoneal cavity of the mouse and diluted with Hank’s balanced salt solution (HBSS) (ascitic fluid:HBSS 1:9). In case of hemorrhagic ascites, the sample was centrifuged and the pellet was incubated with 1ml of red blood cell (RBC) lysis buffer for 5 min at room temperature for optimal lysis of erythrocytes. After centrifugation, the cell pellet was resuspended in HBSS while preserving the original volume and concentration. Cells from ascitic samples were cytospun onto slides, fixed in 4% neutral buffered formalin for 10 min, permeabilized with 0.1% Triton-X/PBS for 10 min, dried and stored at −80 °C. For immunofluorescence staining, slides were thawed, blocked with 5% BSA/PBS solution for 1 h and incubated with primary antibodies overnight at 4 °C, following the immunofluorescence protocol as described above. For detection of CK19^+^ cell clusters, staining against CK19 was performed in cytospun ascitic cells. A cell cluster was characterized as CK19^+^ when 3 or more CK19^+^ cells were detected to be in direct contact.

### 2.9. Evaluation of Macro- and Micro-Metastasis

For the evaluation of macro-metastasis, livers from euthanized animals were removed and observed under the stereoscope for the detection of metastatic foci (minimum diameter = 1 mm). For the evaluation of micro-metastasis, whole livers were serially sectioned with a thickness of 3 μm and a distance of 40 μm between the sections, placed onto slides and H&E stained.

### 2.10. Isolation of Primary Cancer Cells and Primary Cell Culture Establishment

Pancreatic cancer tissue was dissected into small pieces with a scalper and incubated in collagenase D/HBSS (5 mg/mL) (Roche #11088866001) for 30 min at 37 °C. Collagenase D was deactivated after addition of culture medium DMEM (Gibco #41965-039) containing 10% fetal bovine serum (FBS) (Gibco #10270106). Cell suspension was applied successively to cell strainers with 100, 70 and 40 μm pore diameters. Cells were then centrifuged, resuspended in culture medium DMEM/F12 containing GlutaMAX (Gibco #31331028) and B-27 supplement (Gibco #17504-044) and seeded on ultra-low attachment plates (MilliporeSigma #CLS347124EA). After 3 days, cells were harvested and seeded to cell culture dishes (Greiner #664160) with culture medium DMEM (GIBCO #41965-039) containing 10% FBS and 1% L-glutamine (Gibco #25030-024). FibrOut (VWR #10786-022) at a concentration of 0.2% was added to the culture media for 6 days. Cells were cultured for a maximum of 3 passages and were then either harvested for protein isolation or used for invasion and migration assays.

### 2.11. Tumor Necrosis Factor a (TNF-α) Treatment and Nuclear Extraction

Cells were treated with TNF-α at a concentration of 40 ng/mL for one hour and harvested. Nuclear extracts from cells were obtained using the NE-PER™ Nuclear and Cytoplasmic Extraction Kit (ThermoFischer #78833) according to the standard protocol.

### 2.12. Cell Migration and Invasion Assays

Migration assays were performed using inserts of 8 μm pore size and PET membranes (Corning #353097). 5 × 10^4^ cells in serum-free medium were seeded to the inserts. Media containing 10% FBS were added to the bottom well. A total of 24 h later, cells that passed through the membrane were fixed with 4% PFA and stained with DAPI (MilliporeSigma #D9542-5MG). For cell invasion assays, prior to seeding cells, 24-transwell inserts were coated with matrigel (Corning #356237) that was diluted (1:1) with DMEM and allowed to settle for 2 h at 37 °C. For quantitative microscopy, 10 random power fields were captured in 10× magnification. The quantification was performed in ImageJ.

### 2.13. Statistics

Statistical analyses were performed with Graphpad Prism v.8.4.3. Diagrams show arithmetic means and standard deviations (SDs). Student’s *t*-test was used for the comparison of 2 groups (* *p* < 0.05, ** *p* < 0.01, *** *p* < 0.001, **** *p* < 0.0001) while, in case outliers were spotted, a Mann–Whitney test was used (# *p* < 0.05, ## *p* < 0.01, ### *p* < 0.001). One-way analysis of variance (ANOVA) with Tukey’s multiple comparison test was used for the comparison of more than 2 groups, while ANOVA with Dunn’s multiple comparison test was used in case outliers were spotted (# *p* < 0.05, ## *p* < 0.01). For survival analysis, each group was examined by Kaplan–Meier survival estimators, and the survival outcomes were compared using log-rank test (* *p* < 0.05). For the survival analysis of PDAC patients, the human protein atlas (HPA) database was used to compare groups with respect to p65 (RelA) expression and their survival [30,31]. Patients were categorized into two groups according to the fragments per kilobase of transcript per million (FPKM) values of RNA sequencing for p65. According to the HPA website, the cutoff between the two groups yields the maximal difference with respect to survival at the lowest log-rank *p*-value.

## 3. Results

### 3.1. NEMO Is Dispensable for the Development of PDAC but Modulates Progression of Tumors in KPC Mice

NF-κB signaling is regulating the development of different types of cancer [18]. First, we investigated whether differences in the activity of NF-κB signaling also affect the survival of PDAC patients. Thus, we examined the HPA database and classified PDAC patients according to their survival and the expression level of p65 (RelA), an essential component of the transcription factor NF-κB. Patients with higher p65 expression demonstrated a 5-year survival rate of 22%, while patients with low p65 expression exhibited a 5-year survival rate of 41%, indicating that reduced activity of NF-κB signaling is associated with better survival in PDAC (Figure S1).

To analyze the role of NF-κB signaling in PDAC, we crossed mice expressing the Cre recombinase under the Pdx1 promoter with mice carrying one *KRAS^G12D^* allele activated by Cre-mediated recombination, floxed *p53* alleles, and/or a floxed *Ikbkg* allele (homozygous floxed *Ikbkg* in female) (Table 1). Pancreata of mice were analyzed at three different time points: 8 weeks, 12 weeks, or when the mice were in a moribund state and had reached their humane endpoint (HEP) (Figure S2A). For the verification of NEMO ablation, we performed immunoblotting of protein extracts from the pancreata of 8-week-old wild-type (WT) and Pdx1-Cre; p53^fl/fl^; NEMO^fl/fl^ (PNeC) mice (no oncogenic KRAS). As illustrated in Figure S2B, NEMO protein expression was virtually absent in PNeC mice. We did not use KPC and Pdx1-Cre; KRAS^G12D^; p53^fl/fl^; NEMO^fl/fl^ (KPNeC) pancreata to identify NEMO ablation due to the potential development of neoplasia that is accompanied by the infiltration of immune cells and the proliferation of fibroblasts. These immune cells and fibroblasts do not express the Cre-recombinase and, therefore, are normally expressing NEMO, rendering the detection of NEMO ablation inaccurate.

At the time point of 8 weeks, WT, Pdx1-Cre; NEMO^fl/fl^ (NeC), Pdx1-Cre; p53^fl/fl^ (PC) and PNeC pancreata (all missing the oncogenic KRAS) displayed no signs of precancerous lesions, fibrosis or inflammation and were histologically normal (Figure S2C). Pancreata from Pdx1-Cre; KRAS^G12D^ (KC) and Pdx1-Cre; KRAS^G12D^; NEMO^fl/fl^ (KNeC) mice, which harbor the constitutively active KRAS, developed very few precancerous lesions at this early time point, including acinar-ductal metaplasias (ADMs) and PanINs. In addition, low levels of inflammation and fibrosis were present only in the periphery of the precancerous lesions. In general, NEMO deletion did not alter the development of precancerous lesions or the induction of inflammation and fibrosis at the age of 8 weeks (Figure S2C). Our findings are consistent with previous work performed in our lab, where it was demonstrated that NEMO deletion does not affect the development of PanINs at a very young age in KC mice [23].

**Figure 1:**
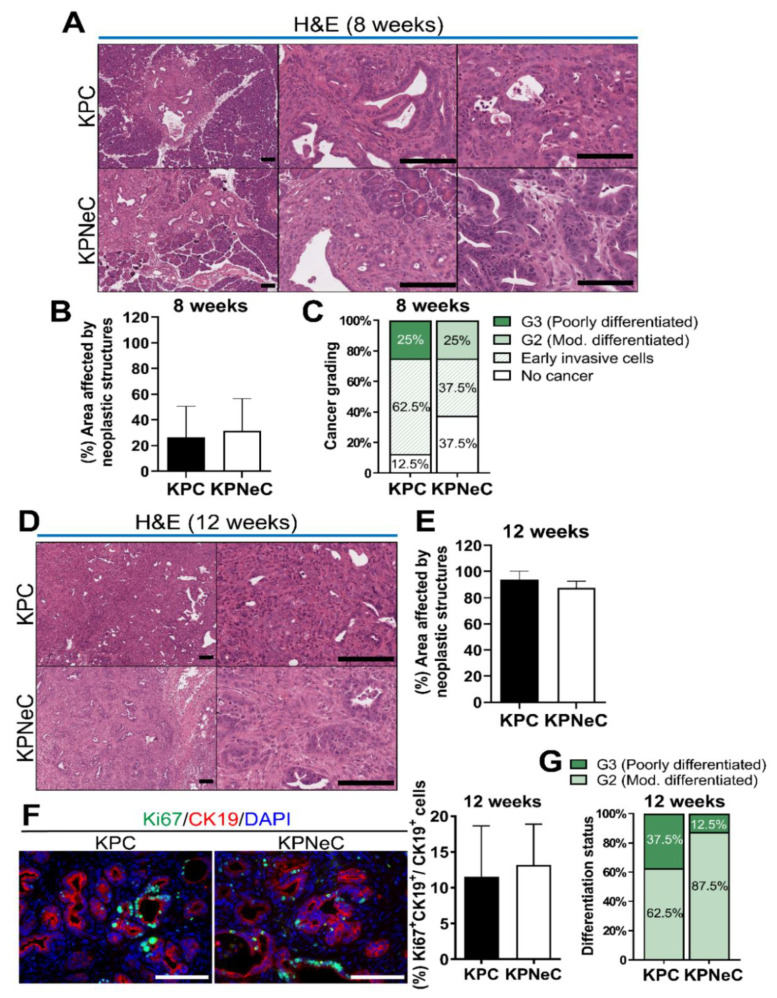
Pancreatic tumor establishment and growth is insusceptible to NEMO ablation in KPC mice. (**A**) H&E staining on pancreatic sections of 8-week-old Pdx1-Cre; KRAS^G12D^; p53^fl/fl^ (KPC) and Pdx1-Cre; KRAS^G12D^; p53^fl/fl^; NEMO^fl/fl^ (KPNeC) mice at different magnifications. Left: General overview of KPC and KPNeC pancreata. Middle: Visualization of early invasive cancer cells. Right: Visualization of G3 PDAC in KPC mice and G2 PDAC in KPNeC mice. Scale bar: 100 μm. (**B**) Percentage of the total abnormal area (precancerous lesions, cancer, fibrosis, inflammation) to the total pancreatic area of pancreatic sections of 8-week-old KPC and KPNeC mice (*n* = 8 mice/group). (**C**) Percentage of cancer development and grading of cancer of 8-week-old KPC and KPNeC mice (*n* = 8 mice/group). (**D**) H&E staining on pancreatic sections of 12-week-old KPC and KPNeC mice at different magnifications. Left: Overview of KPC and KPNeC pancreata. Right: Visualization of full-blown pancreatic cancer on higher magnification. Scale bar: 100 μm. (**E**) Percentage of the total abnormal area to the total pancreatic area of pancreatic sections of 12-week-old KPC and KPNeC mice (*n* = 8 mice/group). (**F**) Left: Visualization of Ki67^+^ and CK19^+^ cells on pancreatic sections of 12-week-old KPC and KPNeC mice. Nuclear staining with DAPI, scale bar: 100 μm. Right: Percentage of Ki67^+^/CK19^+^ cells to the total number of CK19^+^ cells on pancreatic sections (*n* = 6 mice/group). (**G**) Differentiation status of PDAC of 12-week-old KPC and KPNeC mice (*n* = 8 mice/group).

Histological analysis of KPC and KPNeC pancreata, in which the tumor suppressor p53 is deleted to accelerate PDAC formation, revealed the development of neoplastic structures in both groups at the age of 8 weeks (Figure 1A). To identify potential differences in the development of precancerous lesions, we quantified the number of ADMs, low-grade and high-grade PanINs as well as the total field covered by neoplasia. We did not observe any significant difference in the number of any subtype of pancreatic lesions between KPC and KPNeC mice (Figure S2D). In addition, KPC and KPNeC pancreata had approximately 26% and 31% of their normal pancreas replaced by neoplastic structures, respectively, while the majority of the remaining field was still covered by normal acinar cells (Figure 1A,B).

To further investigate this, we stained the exocrine compartment of KPC and KPNeC pancreata using an antibody against α-amylase, a specific marker of acinar cells. We identified that the acinar field was covering, on average, 62% of the KPC pancreata and 56% of the KPNeC pancreata; thus, only a slight difference between the two groups was observed that did not reach statistical significance (Figure S2E).

Next, we examined the histology of KPC and KPNeC pancreata to evaluate the progress of pancreatic cancer. A total of 25% of KPC and 25% of KPNeC mice developed PDAC at the time point of 8 weeks. We also evaluated the differentiation status of these tumors. Using morphological criteria, it is possible to evaluate the resemblance between the tumor and the original tissue. Generally, a higher grade of differentiation of the tumor, which is more similar to the original tissue, is associated with a better prognosis than a lower grade differentiation status [32]. There are four grades of differentiation status, with the most differentiated tumors termed well differentiated (G1), followed by the moderately differentiated (G2), poorly differentiated (G3) and finally the undifferentiated (G4) tumors. We identified that all tumors of KPC mice were poorly differentiated (G3), while all tumors of KPNeC mice were moderately differentiated (G2) (Figure 1A,C). In addition, there were some mice where early invasive cancer cells were detected in their pancreata. Though these cancer cells had already crossed the basement membrane, they were rather scattered single cells but not organized tumor structures, and therefore did not fall in the criteria of full-blown PDAC. We detected 62.5% of the KPC and 37.5% of the KPNeC pancreata with early invasive cancer cells that had not yet developed full-blown PDAC (Figure 1C). These results indicate that KPC and KPNeC mice developed PDAC at the same rate at the time point of 8 weeks. However, tumors in KPC mice developed single invasive cells slightly faster and KPC tumors were less differentiated than the KPNeC tumors.

To track the progression of pancreatic cancer and the fate of the remaining normal pancreas, we analyzed the mice at the time point of 12 weeks. Similar to 8 weeks, WT, NeC, PC and PNeC pancreata (all lacking oncogenic KRAS expression) displayed no signs of abnormal structures (Figure S2F). KC and KNeC pancreata displayed, again, very few ADMs and PanINs, with no difference between these two groups. Slight inflammation and fibrosis were present only in the periphery of the precancerous lesions (Figure S2F).

In contrast, all KPC and KPNeC pancreata were virtually completely replaced by PDAC, infiltrating immune cells and a strong desmoplastic reaction at the time point of 12 weeks (Figure 1D). In particular, neoplastic structures, including inflammation and fibrosis, were covering, on average, 93.6% of KPC and 87.6% of KPNeC pancreata (Figure 1E). The pancreatic (to body) weight ratio of both KPC and KPNeC mice was significantly higher than the pancreatic weight ratio of WT mice (Figure S2G). Next, we investigated whether NEMO deletion regulated the growth of the tumor cells. We stained KPC and KPNeC pancreata for Ki67 and CK19 to identify the proliferating and the neoplastic cells, respectively. From the total population of CK19^+^ cells, we detected 11.6% Ki67^+^ cells in KPC pancreata and 13.2% Ki67^+^ cells in KPNeC pancreata, indicating no overt difference in the proliferation of neoplastic cells between the two groups (Figure 1F).

Further, we analyzed the differentiation status of the tumors. A total of 37.5% of the KPC mice developed a poorly differentiated tumor (G3) and the rest of the KPC mice developed a moderately differentiated tumor (G2). In contrast, only 12.5% of the KPNeC mice developed a poorly differentiated tumor (G3), while the rest of the KPNeC mice developed a moderately differentiated tumor (G2) (Figure 1G). These results revealed that, at the time point of 12 weeks, KPC mice developed less differentiated tumors than KPNeC mice, indicating that NEMO deletion may support an increased differentiation status of PDAC.

Next, we compared the total remaining exocrine compartment between KPC and KPNeC pancreata at the time point of 12 weeks. Immunofluorescence staining against α-amylase revealed that only a few acinar cells were still present in both groups. Specifically, acinar cells were covering 10% of the KPC and 13% of the KPNeC pancreata, indicating that the exocrine compartment of both KPC and KPNeC pancreata was seriously reduced (Figure S2H).

Immune reaction in PDAC patients is localized to the juxtatumoral stromal compartment [33]. While immune cells are attracted towards pancreatic cancer cells, the majority of them cannot infiltrate the tumor due to the strong desmoplastic reaction and only a few can reach their target [33]. To investigate the localization of the immune cells and the extent of desmoplasia in KPC and KPNeC pancreata, we stained tissue slides against the pan-leukocyte marker CD45 or performed AZAN Trichrome staining. As illustrated in Figure S2I, most immune cells were concentrated in the periphery of the tumor in both groups, while only a few of the immune cells could actually invade and reach the core of the tumor. In addition, quantification of the fibrotic area by AZAN staining did not reveal any overt difference between the two groups (Figure S2J). The abovementioned results indicate that all KPC and KPNeC mice developed PDAC with 100% penetrance at the age of 12 weeks, with no significant differences in their immune and fibrotic reaction.

### 3.2. Pancreas-Specific NEMO Ablation Improves Survival in KPC Mice

**Figure 2:**
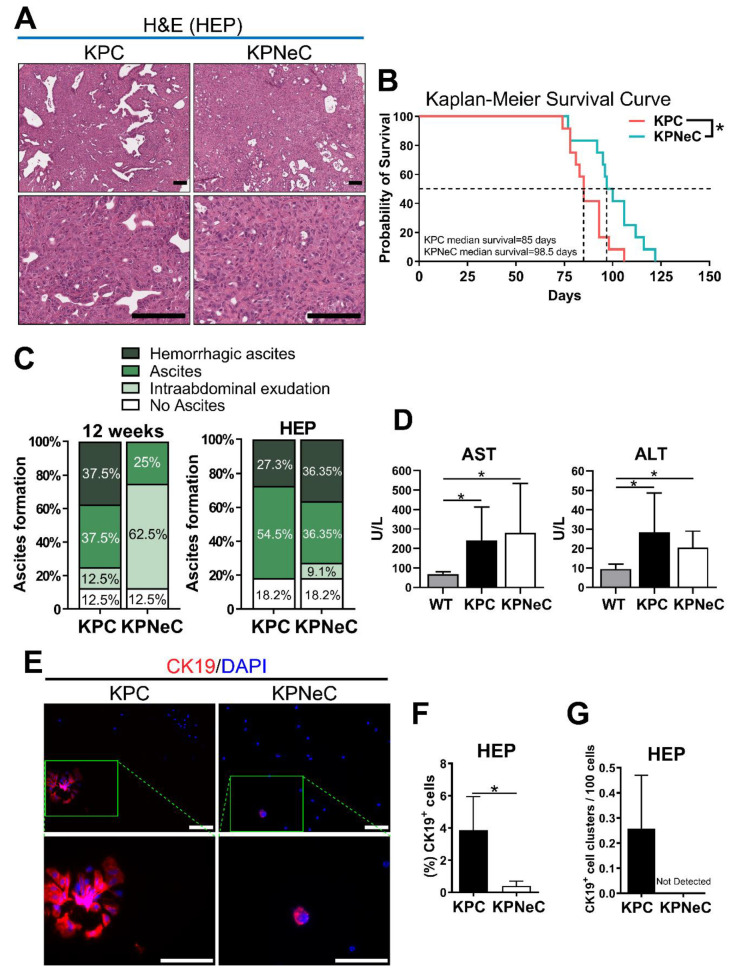
Pancreas-specific NEMO ablation increases the survival rate of KPC mice. (**A**) H&E staining on pancreatic sections of HEP-analyzed KPC and KPNeC mice on different magnifications. Scale bar: 100 μm. (**B**) Kaplan–Meier survival curve for KPC (coral line) and KPNeC (cyan line) mice (*n* = 12 mice/group; log-rank test: * *p* < 0.5). (**C**) Grading of ascites development in 12-week-old and HEP-analyzed KPC and KPNeC mice (*n* = 8 mice/group). (**D**) Quantification of aspartate transaminase (AST) and alanine transaminase (ALT) levels in the serum of the indicated groups. (U/L = Units/Liter; serum from KPC and KPNeC mice was extracted at their HEP; serum from WT mice was extracted at the age of 12 weeks; *n* ≥ 7 mice/group; student’s *t*-test: * *p* < 0.5). (**E**) Visualization of CK19^+^ ascitic cells isolated from HEP-analyzed KPC and KPNeC mice. Nuclear staining with DAPI, scale bar: 100 μm. (**F**) Percentage of CK19^+^ cells to the total number of ascitic cells (*n* = 3 mice/group; student’s *t*-test: * *p* < 0.5). (**G**) Percentage of CK19^+^ cell clusters per 100 ascitic cells (*n* = 3 mice/group).

Next, we evaluated the effect of NEMO ablation on the survival of the mice. Moribund KPC and KPNeC mice developed PDAC with a 100% PDAC penetrance and no difference in their pancreatic weight (Figure 2A and Figure S3A). These mice were characterized by breathing difficulties, reduced vigilance and limited mobility, while they occasionally developed jaundice. Importantly, Kaplan–Meier survival analysis revealed an extended lifespan of KPNeC mice (median survival of KPNeC mice = 98.5 days) compared to KPC mice (median survival of KPC mice = 85 days) (Figure 2B). Therefore, we investigated possible causes that affected their survival. Notably, we observed that KPC mice developed ascites much earlier than KPNeC mice.

Malignant ascites, an accumulation of fluid with cancer cells in the abdominal cavity, is a common complication in human patients with pancreatic cancer [34]. Causes of malignant ascites development include peritoneal carcinomatosis, lymphatic vessel obstruction and portal hypertension. In addition, malignant ascites regularly appears in cases where pancreatic cancer has already metastasized and is generally associated with very poor prognosis [35]. Most importantly, the KPC mouse model is able to recapitulate the development of ascites [36]. In our study, 75% of the KPC mice developed ascites at the time point of 12 weeks, with 50% of these cases being hemorrhagic. Conversely, only 25% of the KPNeC mice developed ascites at the same time point, and all of them were non-hemorrhagic (Figure 2C). Interestingly, 62.5% of the KPNeC mice displayed slight intraabdominal exudation, indicating potential fluid accumulation and ascites development at a later time point. In contrast, analysis at the HEP revealed no overt difference between the two groups, with 81.8% of the KPC and 72.7% of the KPNeC mice having developed ascites at the moribund state (Figure 2C). Ascites development was not likely a result of peritoneal carcinomatosis since no peritoneal metastasis was observed. However, elevated levels of aspartate transaminase (AST) and alanine transaminase (ALT) were detected in the serum of the mice, indicating that portal hypertension or liver injury could have been a cause of ascites (Figure 2D).

To evaluate the malignancy of the formed ascites, we isolated cells from the accumulated fluid in the abdomen of HEP-analyzed mice and stained them for CK19 to identify pancreatic cancer cells. We could detect significantly more CK19^+^ cells in the ascites isolated from KPC mice than from KPNeC mice, indicating that NEMO/NF-κB signaling is supporting the detachment of pancreatic tumor cells from the primary tumor or the formation of clusters in the ascites (Figure 2E,F). We further noticed that the ascitic CK19^+^ cells from KPC mice tended to cluster, a feature that has been associated with increased metastatic properties [37]. As illustrated in Figure 2G, we detected CK19^+^ cell clusters in all cases of ascites deriving from KPC mice, whereas, in the absence of NEMO, we did not observe any CK19^+^ cell clusters. Staining of CD45 indicated that immune cells were abundant in ascites (Figure S3B).

### 3.3. Pancreas-Specific NEMO Ablation Reduces the Metastasis Rate in KPC Mice and Blocks EMT

**Figure 3:**
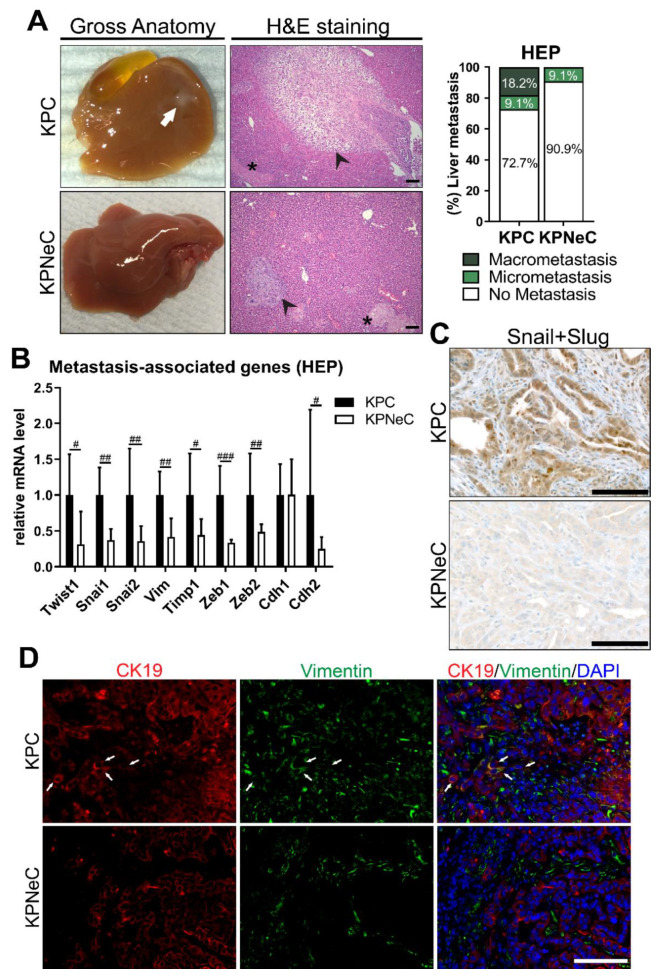
Pancreas-specific NEMO ablation abrogates macro-metastasis in KPC mice and blocks epithelial–mesenchymal transition (EMT). (**A**) Left: Visualization of gross anatomy of the liver and H&E staining on liver sections of HEP-analyzed KPC and KPNeC mice. Arrow: Macro-metastasis. Arrowhead: Micro-metastasis. Asterisk: necrosis. Scale bar: 100 μm. Right: Quantification of liver macro- and micro-metastasis of HEP-euthanized KPC and KPNeC mice. (*n* = 11 mice/group). (**B**) Quantitative RT-PCR for the expression of the indicated transcripts in pancreatic tissue of HEP-analyzed KPC and KPNeC mice, given relative to KPC mice, which were set to 1 (*n* ≥ 6 mice/group; Mann–Whitney test: # *p* < 0.05, ## *p* < 0.01, ### *p* < 0.001). (**C**) Visualization of Snail+Slug staining on pancreatic sections of 12-week-old KPC and KPNeC mice. Scale bar: 100 μm. (**D**) Visualization of CK19^+^ and Vimentin^+^ cells on pancreatic sections of 12-week-old KPC and KPNeC mice. Nuclear staining with DAPI. Arrow: Indicative CK19^+^/Vimentin^+^ cells, scale bar: 100 μm.

These findings indicated that whereas NEMO/NF-κB signaling is not crucial for primary tumor development in the KPC model, disease progression and most likely metastasis is altered. Therefore, we investigated whether NEMO ablation affects the metastatic properties of pancreatic cancer cells and analyzed the livers of KPC and KPNeC mice at their HEP. Examination of the livers revealed that 18.2% of the KPC mice developed liver macro-metastasis, while, in the absence of NEMO, no mice were detected with liver macro-metastasis (Figure 3A). Interestingly, we observed hepatocellular necrosis in the livers of KPC and KPNeC mice, possibly as a result of the pressure from the enlarged tumor towards the liver (Figure 3A). To identify the presence of liver micro-metastasis, we cut the whole liver into sections. Examination of the liver histology revealed established areas of metastatic tumor in 27% of KPC mice. Conversely, in the absence of NEMO, livers were free of cancer cells with the exception of the liver of one mouse, where a small area containing a few cancer cells was detected (Figure 3A).

A widely accepted theory of how cancer cells metastasize to distant organs is the activation of the EMT program [38]. EMT involves an alteration of the gene expression program of the epithelial cells towards a program typical for mesenchymal cells. It changes the shape of cancer cells, the composition of their adhesion molecules as well as their migrating/invasive properties, which subsequently favors metastasis. Previous studies in the field showed that the NF-κB pathway is essential for the regulation of EMT in various types of cancer, including PDAC [38,39,40,41]. Therefore, we analyzed the expression level of EMT-associated genes in KPC and KPNeC mice at their HEP. With respect to transcription factors associated with EMT, we observed that NEMO deletion strongly reduced the RNA levels of *Twist1*, *Snai1* and *Snai2*. In addition, *Cdh2* and *Vimentin*, two mesenchymal cell-markers, were also strongly downregulated in KPNeC mice, indicating that NEMO deletion diminished the EMT signaling (Figure 3B). We also quantified the expression of *Cdh1*, a marker of epithelial cells, but, interestingly, we did not observe any difference in its expression in the absence of NEMO. Finally, *tissue inhibitor of metalloproteinase 1 (Timp1)*, which is necessary for the establishment of a premetastatic niche in the liver and favors the establishment of macro-metastasis [42], was downregulated in the absence of NEMO (Figure 3B).

To further confirm the down regulation of the EMT program, we performed immunostaining in sections of KPC and KPNeC pancreata and examined the protein expression and the localization of EMT-associated markers. With respect to the Snail (Snai1) and Slug (Snai2) transcription factors, NEMO deletion strongly reduced their expression as well as their translocation to the nucleus (Figure 3C). In addition, we could detect CK19^+^/Vimentin^+^ cells in KPC pancreata as a result of the EMT process, while the absence of NEMO diminished the number of these cells (Figure 3D). Similar to the results of the transcriptional analysis, E-cadherin expression was not regulated in the absence of NEMO (Figure S4).

### 3.4. NEMO Ablation Diminishes the Migrating and Invasive Properties of KPC Cells Ex Vivo

**Figure 4:**
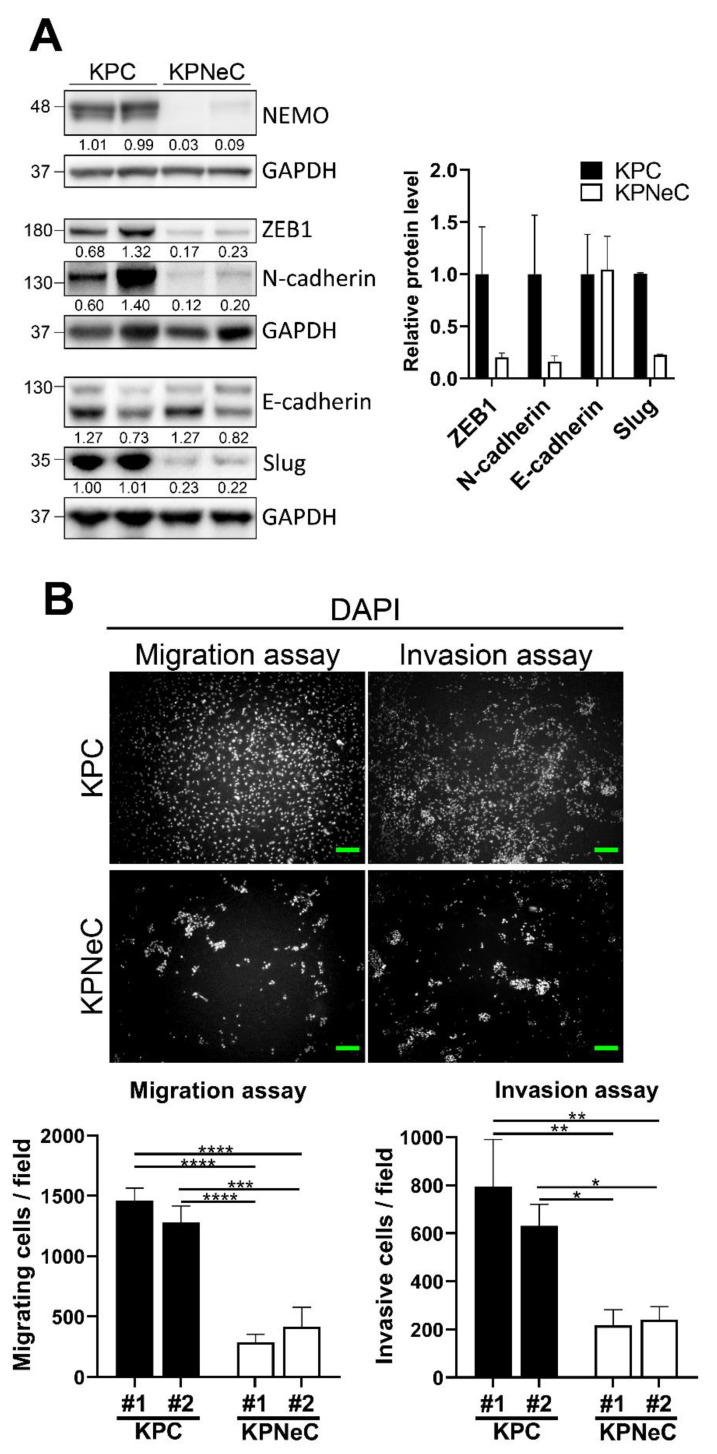
NEMO ablation diminishes the migrating/invasive properties of KPC cells ex vivo. (**A**) Left: Western blot analysis of primary cancer cell protein extracts from KPC and KPNeC mice. GAPDH was used as a loading control. Right: Quantification of the Western blot analysis. The diagrams show the quantification of the ZEB1/GAPDH, N-cadherin/GAPDH, E-cadherin/GAPDH and Slug/GAPDH ratios, given relative to KPC cells, which were set to 1 (*n* = 2/group). (**B**) Top: Cell migration and invasion assays of cancer cells harvested from cell cultures derived from 2 different KPC and 2 different KPNeC mice. Scale bar: 100 μm. Bottom: Quantification of migrating and invasive cells derived from KPC and KPNeC mice after 24 h; student’s *t*-test: * *p* < 0.05, ** *p* < 0.01, *** *p* < 0.001, **** *p* < 0.0001.

NEMO deletion hampered the activation of the EMT program and substantially reduced the liver metastasis rate in KPC mice. To analyze whether these observed changes in gene expression alter the invasive properties of KPC and KPNeC cells ex vivo, we isolated cancer cells from their respective primary tumors and established primary cancer cell cultures. Firstly, we verified that the process of clearing the primary cancer cell population of fibroblasts and immune cells is successful by immunoblotting protein extracts of KPNeC primary cultures against NEMO. While pancreatic cancer cells derived from KPNeC mice do not express NEMO, fibroblasts and immune cells lack Cre-recombinase and express NEMO at normal levels. Immunoblotting revealed the absence of NEMO (Figure 4A); thus, we could verify that the cell culture populations were free of fibroblasts and immune cells.

Next, we evaluated the inhibition of the NF-κB signaling in the absence of NEMO. We first stimulated primary cancer cells of KPC and KPNeC pancreata with TNF-α. We then performed nuclear protein extraction and examined the level of nuclear p65 by Western blot. While there was a strong accumulation of p65 in the nuclear fraction of KPC cells after TNF-α stimulation, this translocation was severely reduced in KPNeC cells (Figure S5A).

We then performed immunoblot analysis using whole protein extracts from KPC and KPNeC primary cultures to compare the expression level of EMT-associated markers. Notably, ZEB1, N-cadherin (*Cdh2*) and Slug (*Snai2*), all EMT-associated markers, were downregulated in the absence of NEMO, while E-cadherin (*Cdh1)* expression was preserved at a similar level in the absence of NEMO (Figure 4A). These results indicate that the downregulation of EMT-associated markers in the absence of NEMO is preserved ex vivo.

Finally, we cultured primary cancer cells derived from two different KPC and two different KPNeC mice and performed functional assays to evaluate the invasive and migrating properties of the cell culture populations. Strikingly, NEMO deletion hampered these two properties of the primary PDAC cells. Specifically, KPNeC cancer cells exhibited a 4-fold reduction in the number of migrating cells in the cell-migration assays. In addition, a 3-fold reduction in the number of invasive cells in the cell-invasion assays was observed in the absence of NEMO (Figure 4B). To verify that the proliferation rate of the primary cancer cells did not affect the outcome of the invasion and migration assays, we compared their doubling time but observed no significant difference between the groups (Figure S5B). These findings revealed that blocking the NF-κB signaling inhibited the invasive/migrating properties of pancreatic cancer cells both in vivo and ex vivo.

## 4. Discussion

NF-κB signaling, which regulates critical cellular functions, has been associated with the development of carcinoma as well as the establishment of metastasis [18,43]. In the present study, we investigated the role of NF-κB in these two processes using a genetic mouse model of the pancreas-specific expression of oncogenic KRAS combined with the deletion of the tumor suppressor p53 (KPC mouse model). This model is widely used and has been well characterized, although there have been described diversities in metastasis depending on the inactivation method of p53 (point mutation or deletion) [44,45]. Nonetheless, this model generally results in rapid tumor development, while a percentage of these mice develop metastasis at their HEP [45]. To blunt the conventional NF-κB pathway, KPC mice were crossed to NEMO-floxed mice, allowing for the pancreas-specific deletion of NEMO. We discovered that NEMO deletion had little effect on the development of the primary tumor but altered the differentiation status of the tumors. Importantly, NEMO deletion extended the survival of the mice, accompanied by a significant reduction in the metastatic properties of the cancer cells.

The role of NF-κB in the development of cancer has been meticulously studied in the past. NF-κB regulates inflammation, one of the hallmarks of cancer; thus, it was first thought that the inhibition of this pathway could be used as a treatment against different types of cancer [46]. However, although the inhibition of NF-κB can potentially hinder the development of some types of cancer, including PDAC, there have been studies underscoring the fact that the role of NF-κB can be highly context- and cell-type-dependent and that the blocking of this pathway can result in opposing results [18,23,47]. For instance, while constitutive activation of the NF-κB pathway supports hepatocellular carcinoma development and progression, the absence of NF-κB signaling in liver cells also significantly promotes liver cancer development [48]. Therefore, the role of NF-κB has to be analyzed depending on each cancer type specifically but also depending on the circumstances and conditions that supported the development of cancer. Our study highlights that NEMO is dispensable for the development of PDAC in KPC mice. Comparison of the proliferation rate of neoplastic cells and the number of the precancerous lesions did not reveal any difference between KPC and KPNeC mice.

While previous studies have reported that NF-κB regulates the progression of pancreatic precancerous lesions, it is important to note that these studies used a different approach by focusing on the KC model [22,23,49]. KC mice develop low-grade PanINs, which must accumulate additional mutations to advance towards high-grade PanINs. The accumulation of the additional mutations is regulated by cellular responses that can be controlled by the conventional NF-κB pathway. For instance, we have previously shown that NEMO deletion in KC mice reduces the expression of pro-inflammatory and fibrogenic chemokines that are regulated by NF-κB. Consequently, diminished inflammatory and fibrotic responses result in a reduction in the total PanIN1 lesions by 93% [23]. Comparably, the deletion of IKK2 in KC mice strongly reduces the expression of Notch target HES1 and subsequently increases the expression of the anti-inflammatory nuclear receptor PPAR-γ. This anti-inflammatory response reduces the development of PanINs, although to a lesser extent than NEMO deletion [25]. These studies all indicate the importance of NF-κB activities in sustaining a tumor-promoting inflammatory microenvironment. In an opposite direction from the previous studies, RelA deletion in KC mice accelerates the progression of low-grade PanINs by inhibiting the oncogene-induced senescence program, which is normally active in low-grade PanINs [49]. All of the aforementioned studies share the aspect of regulating the progression of low-grade to high-grade PanINs. In contrast, in our study, we used the KPC mouse model, which rapidly develops PDAC. It is necessary to emphasize that KPC mice develop PanINs carrying a mutational background with constitutively active KRAS and no expression of p53, which already resembles the background of high-grade PanINs. The shorter latency of tumor development in the KPC mouse model may also diminish the importance of NF-κB in maintaining the tumor-promoting inflammatory microenvironment. Although a deletion of IKK2 in another PDAC mouse model involving an inactivation of Ink4a/Arf shows a complete rescue up to 12 months [22], NEMO deletion only slightly extends the lifespan of KPC mice. It also has little effect with respect to the development of early invasive cells and generally does not alter the development of PDAC. Inactivation of p16 (Ink4a/Arf) usually starts at an earlier stage of PanIN lesions (PanIN2), while, as mentioned, inactivation of p53 occurs in a more advanced stage (PanIN3) during PanIN progression and PDAC development [50]. Therefore, although inactivation of p16 or p53 both can lead to accelerated PDAC development, it is possible that p53 inactivation can drive the development of an advanced type of tumor within a shorter time and cannot be completely rescued by NF-κB inhibition.

While NEMO deletion did not significantly influence the development of PDAC, it supported the development of more differentiated tumors. Analysis at the time point of 8 weeks revealed that tumors in KPC mice were poorly differentiated, while tumors in KPNeC mice were moderately differentiated. In a similar fashion, analysis at the time point of 12 weeks revealed that there were more KPNeC mice than KPC mice developing more differentiated PDAC.

Further, our study demonstrates that NEMO deletion prolonged the median survival of the mice. Importantly, examination of the livers revealed that NEMO deletion completely abrogated liver macro-metastasis, despite the fact that KPNeC mice had an extended period to develop metastasis due to their extended lifespan. In addition, NEMO deletion strongly reduced the invasive and migrating properties of KPC-derived cancer cells ex vivo, supporting the hypothesis that NF-κB is essential for metastasis in PDAC.

Interestingly, previous studies demonstrated that, depending on the inactivating method of p53, different outcomes with respect to the establishment of metastasis can be observed [44,45]. In our study, we observed results consistent with the study of Bardeesy et al. [45]. Deletion of p53 promoted the development of PDAC and led to the establishment of liver metastasis in 27% of the KPC mice.

The NF-κB pathway has been shown in the past to be a critical regulator of EMT [39]. Blocking the NF-κB pathway diminishes the mesenchymal transition of cancer cells in different types of cancer, including breast, hepatocellular and non-small cell lung carcinoma [38,39,51,52]. In line with the existing literature, we observed that NEMO deletion led to the downregulation of EMT transcription factors and mesenchymal markers in pancreatic tumors as well as to a reduction in the expression level of EMT markers ex vivo. Interestingly, we observed that the expression of E-cadherin, an epithelial cell marker, was preserved during EMT in the absence of NEMO. These findings are consistent with previous studies describing that E-cadherin downregulation may not be necessary for the development and progression of EMT in cancer cells [53,54,55].

Our findings partially contrast with a previous study that reported EMT as dispensable for the establishment of metastasis in PDAC [56]. Through genetic manipulation, either *Twist1* or *Snai1* were knocked out in KPC mice (with point-mutation p53 inactivation), yet liver metastasis was still observed. However, these results can also be interpreted in a way that EMT is a complex multi-factorial procedure; hence, the deletion of an individual regulator may not block the whole process. Alternatively, consistent with our results, previous studies reported that NF-κB is crucial for the mesenchymal and metastatic properties of pancreatic cancer cells [41,57,58,59]. For instance, the expression of dominant negative IκBα in human PDAC cell lines abolished the development of liver metastasis when the cells were orthotopically injected in nude mice [57]. In a similar manner, the pharmacological inhibition of NF-κB strongly reduced the invasive and migrating properties of IL-18-over-expressing pancreatic cancer cells [58].

Our study also reveals that NEMO deletion strongly reduced the expression of TIMP1 in the pancreata of KPC mice. TIMP1 is a matrix metalloprotease that has been implicated in different types of cancer, and its expression is upregulated upon the activation of the conventional NF-κB pathway [60,61,62]. In addition, a recent study demonstrated that TIMP1 is secreted by premalignant pancreatic lesions, and, through blood circulation, it activates hepatic stellate cells that subsequently promote the establishment of a premetastatic niche in the liver [42]. In our study, we observed liver macro-metastasis only in KPC mice, whereas, in the absence of NEMO, no macro-metastasis was observed. However, we detected one case of a KPNeC mouse where a few cells were detected in a small field of its liver. It is possible that these cancer cells could manage to migrate to the liver in the absence of NEMO, although they were not able to establish macro-metastasis due to the lack of the supporting premetastatic niche. Nevertheless, it is yet unclear to what extent the different consequences of reduced EMT and reduced TIMP1 expression affected the observed reduction in metastasis.

Finally, we identified that NEMO deletion strongly reduced the number of mice developing ascites at the time point of 12 weeks. Analysis of the cell composition in the ascitic fluid revealed less CK19^+^ cells in KPNeC mice, indicating that cancer cells are less likely to detach from the primary tumor when NEMO is absent. Of note, we discovered that ascitic cancer cells from KPC mice tended to detach from the primary tumor in clusters or form clusters in ascites, while, in KPNeC mice, cancer cells in ascites were single cells. In line with our results, it was previously described that cancer cell clusters in the blood of KPC mice are characteristic of enhanced metastatic potential. These cell clusters migrate through the bloodstream towards distinct organs protected from the hazardous environment, while at the same time they support the establishment of metastasis [37].

## 5. Conclusions

The development of metastasis is a crucial factor determining the lifespan of pancreatic cancer patients. We found that NEMO deletion inhibited the development of liver macro-metastasis in KPC mice. Further, we detected that, in the absence of NEMO, mice exhibited a prolonged lifespan by 16%. Interestingly, KPNeC mice were also characterized by a reduced likelihood of developing ascites compared to KPC mice. Our study also reveals that there was no difference in the establishment of pancreatic cancer between NEMO-expressing and NEMO-ablated KPC mice, suggesting that the NF-κB pathway may be dispensable for the progression of high-grade PanINs towards pancreatic cancer on the background of ablated p53. Conclusively, our study provides evidence for a detrimental role of the conventional NF-κB pathway in the survival of KPC mice and supports the establishment of metastasis. These findings underscore the fact that therapeutic approaches against NF-κB should be considered for the treatment of PDAC.

## Figures and Tables

**Table 1:** Nomenclature of the mouse models used in the study.

Mouse Model	Genotype
WT	---
NeC	Pdx1-Cre; NEMO^fl/fl^
PC	Pdx1-Cre; p53^fl/fl^
PNeC	Pdx1-Cre; p53^fl/fl^; NEMO^fl/fl^
KC	Pdx1-Cre; LSL-KRAS^G12D^
KNeC	Pdx1-Cre; LSL-KRAS^G12D^; NEMO^fl/fl^
KPC	Pdx1-Cre; LSL-KRAS^G12D^; p53^fl/fl^
KPNeC	Pdx1-Cre; LSL-KRAS^G12D^; p53^fl/fl^; NEMO^fl/fl^

## Data Availability

The data are available on request from the corresponding authors.

## Supplementary Materials

The following are available online at https://www.mdpi.com/article/10.3390/cancers13184541/s1, Figure S1. Kaplan-Meier survival analysis for low expression (blue line) and high expression (pink line) of RelA (p65) in PDAC patients. The figure is derived and modified from the human protein atlas (HPA) website; Figure S2. Analysis of pancreata of 8-week-old and 12-week-old mice; Figure S3. Analysis of KPC and KPNeC mice at their HEP; Figure S4. Visualization of CK19^+^ and E-cadherin^+^ cells on pancreatic sections of 12-week-old KPC and KPNeC mice. Nuclear staining with DAPI, scale bar: 100 μm; Figure S5. NEMO deletion inhibits NF-κB signaling in KPC primary cancer cells; Table S1. Primers and primary antibodies used in the study.

## Appendix A

Tissue was either snap-frozen in liquid nitrogen, or formalin fixed and paraffin embedded (FFPE). Snap-frozen tissue was cut using a conventional cryotome with a thickness of 4 μm and stored at −80 °C for later staining. FFPE tissue was cut, placed onto slides with a thickness of 3 μm and rehydrated before staining. For H&E staining, standard protocols were used.

The antigen was retrieved with heat-mediated antigen retrieval by incubation with citrate buffer at pH = 6 in a pressure cooker for 10 min (only for FFPE tissue). Sections were washed in distilled water, incubated in 3% hydrogen peroxide (only for immunohistochemistry), blocked with 5% bovine serum albumin (BSA)/phosphate-buffered saline (PBS) solution for 1 h and incubated with primary antibodies overnight at 4 °C. For immunofluorescence, the next day, sections were incubated with secondary antibodies coupled with AlexaFluor-488, Cy3, AlexaFluor-594 or AlexaFluor-647 for 1 h at room temperature and then incubated in DAPI (SigmaAldrich #D9542, dissolved in PBS, c = 300 nM) for 5 min. For immunohistochemistry, the next day, sections were serially incubated with biotinylated secondary antibody for 30 min, streptavidin for 30 min, AEC+ High Sensitivity Substrate Chromogen (DAKO #K3461) for 4 min and counterstained with Hematoxylin for 20 s.

Heidenhain’s azocarmine aniline blue stain (AZAN) staining: For the visualization of collagen, formalin-fixed sections were stained using the Heidenhain’s AZAN staining kit (Morphisto #12079). Slides were incubated for 30 min at 60 °C, deparaffinized by incubation in xylene for 5 min, then serially incubated 2 times to 100% ethanol for 5 min, then to aniline-alcohol for 5 min and rinsed in distilled water. Tissue was stained in azocarmine G 0.1% for 45 min at 60 °C, washed with distilled water, incubated in aniline-alcohol for 5 min and then to acetic acid alcohol 1% for 1 min. Then, the slides were rinsed in distilled water, incubated in phosphotungstic acid 5% for 90 min, stained in Aniline Blue-Orange G for 1 h, rinsed in distilled water, incubated in 100% ethanol for 5 min, then in xylene for 5 min and finally mounted with Entellan mounting medium.
