# Supplementary material for: Deletion of NEMO Inhibits EMT and Reduces Metastasis in KPC Mice
Source: Cancers (Basel). 2021 Sep 10;13(18):4541. doi: 10.3390/cancers13184541 (PMC8471477; doi:10.3390/cancers13184541)
Supplement: Supplementary file 1 [file cancers-13-04541-s001.zip › cancers-1350243-supplementary.pdf]

cancers

MDPI

Article

Deletion of NEMO Inhibits EMT and Reduces Metastasis in KPC Mice

Miltiadis Tsesmelis [1], Kanishka Tiwary [2], Katja Steiger [3], Nadine Sperb [1], Melanie Gerstenlauer [1], Uta Manfras [1], Harald J. Maier [1,4], Patrick C. Hermann [2], Lap Kwan Chan [1,5,6,*] and Thomas Wirth [1,*]

Supplementary Materials

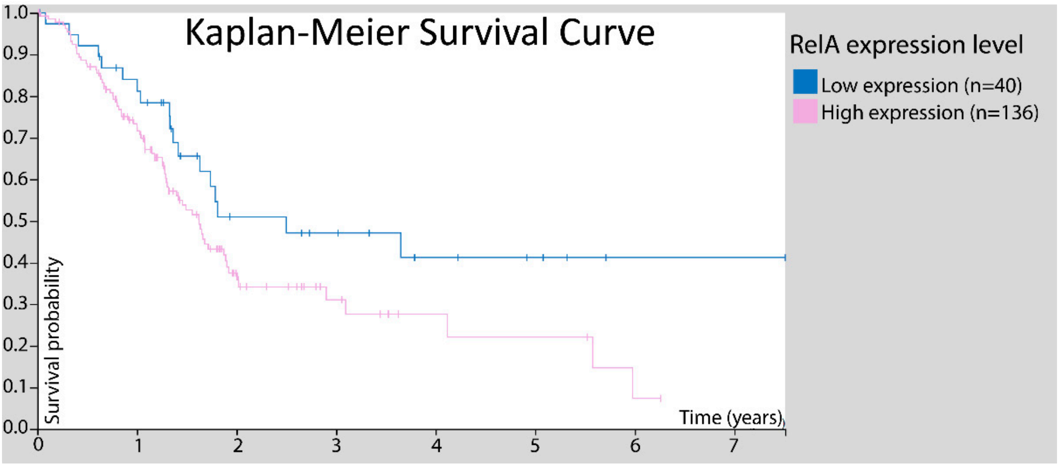

Figure S1. Kaplan-Meier survival analysis for low expression (blue line) and high expression (pink line) of RelA (p65) in PDAC patients. The figure is derived and modified from the human protein atlas (HPA) website (v20.proteinatlas.org/ENSG00000173039-RELA/pathology/pancreatic+cancer).

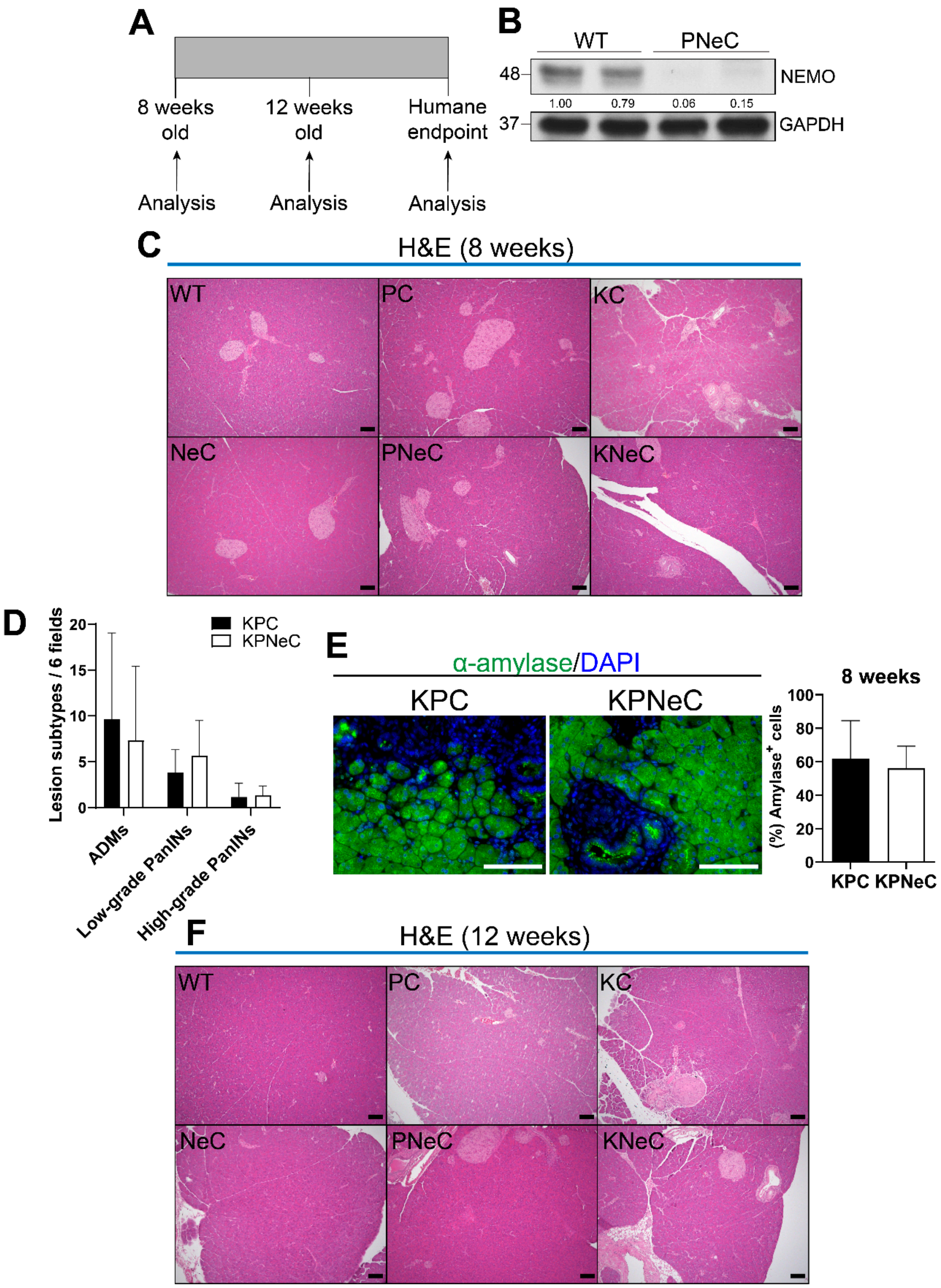
A
8 weeks old
12 weeks old
Humane endpoint
Analysis
Analysis
Analysis
B
WT
PNeC
48
37
NEMO
GAPDH
1.00
0.79
0.06
0.15
C
H&E (8 weeks)
WT
PC
KC
NeC
PNeC
KNeC
D
Lesion subtypes / 6 fields
20
15
10
5
0
KPC
KPNeC
ADMs
Low-grade PanINs
High-grade PanINs
E
α-amylase/DAPI
KPC
KPNeC
8 weeks
(%) Amylase+ cells
100
80
60
40
20
0
KPC KPNeC
F
H&E (12 weeks)
WT
PC
KC
NeC
PNeC
KNeC

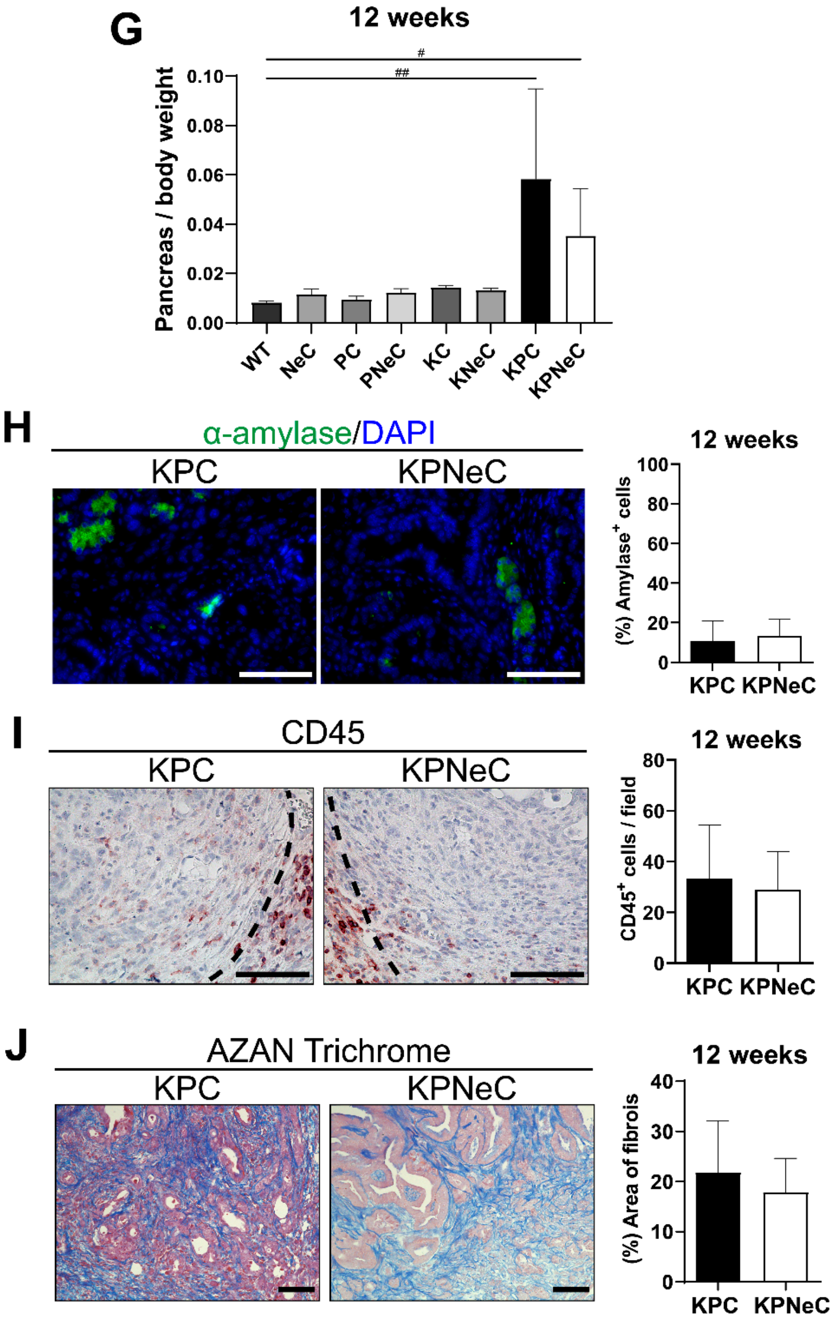

Figure S2. Analysis of pancreata of 8-week-old and 12-week-old mice. (A) Schedule of analysis of the different mouse models at the time point of 8 weeks, 12 weeks or at their humane endpoint (HEP). (B) Western blot analysis of pancreatic protein extracts from wild-type (WT) and Pdx1-Cre;p53$^{fl/fl}$;NEMO$^{fl/fl}$ (PNeC) mice. GAPDH was used as a loading control. (C) H&E staining on pancreatic sections of 8-week-old WT, Pdx1-Cre;NEMO$^{fl/fl}$ (NeC), Pdx1-Cre;p53$^{fl/fl}$ (PC), PNeC, Pdx1-Cre;KRAS^{G12D} (KC) and Pdx1-Cre;KRAS^{G12D};NEMO$^{fl/fl}$ (KNeC) mice. (D) Quantification of acinar-ductal metaplasias (ADMs), low-grade and high-grade PanINs on pancreatic sections of 8-week-old KPC and KPNeC mice (N=6 mice/group). (E) Left: Visualization of α-amylase$^+$ cells on pancreatic sections of 8-week-old KPC and KPNeC mice. Nuclear staining with DAPI, scale bar: 100 µm. Right: Percentage of field covered by α-amylase$^+$ cells to the field covered by the total number of cells (N=6 mice/group). (F) H&E staining on pancreatic sections of 12-week-old WT, NeC, PC, PNeC, KC and KNeC mice. (G) Quantification of pancreatic weight to body weight ratio for the indicated groups (KPC and KPNeC groups: N=8 mice/group, rest of the groups: N≥3 mice/group; one-way analysis of variance with Dunn's multiple comparison test: #p<0.05, ##p<0.01). (H) Left: Visualization of α-amylase$^+$ cells on pancreatic sections of 12-week-old KPC and KPNeC mice. Nuclear staining with DAPI, scale bar: 100 µm. Right: Percentage of field covered by α-amylase$^+$ cells to the field covered by the total number of cells (N=6 mice/group). (I) Left: Visualization of CD45$^+$ cells on pancreatic sections of 12-week-old KPC and KPNeC mice. Scale bar: 100 µm. Dashed line: Separation of immune cells from rest of the tumor, scale bar: 100 µm. Right: Quantification of CD45$^+$ cells on pancreatic sections (N=6 mice/group). (J) Left: Visualization of fibrosis by Heidenhain's azocarmine aniline blue stain (AZAN) staining on pancreatic sections of KPC and KPNeC mice, scale bar: 100 µm. Right: Quantification of fibrotic area (N≥4 mice/group).

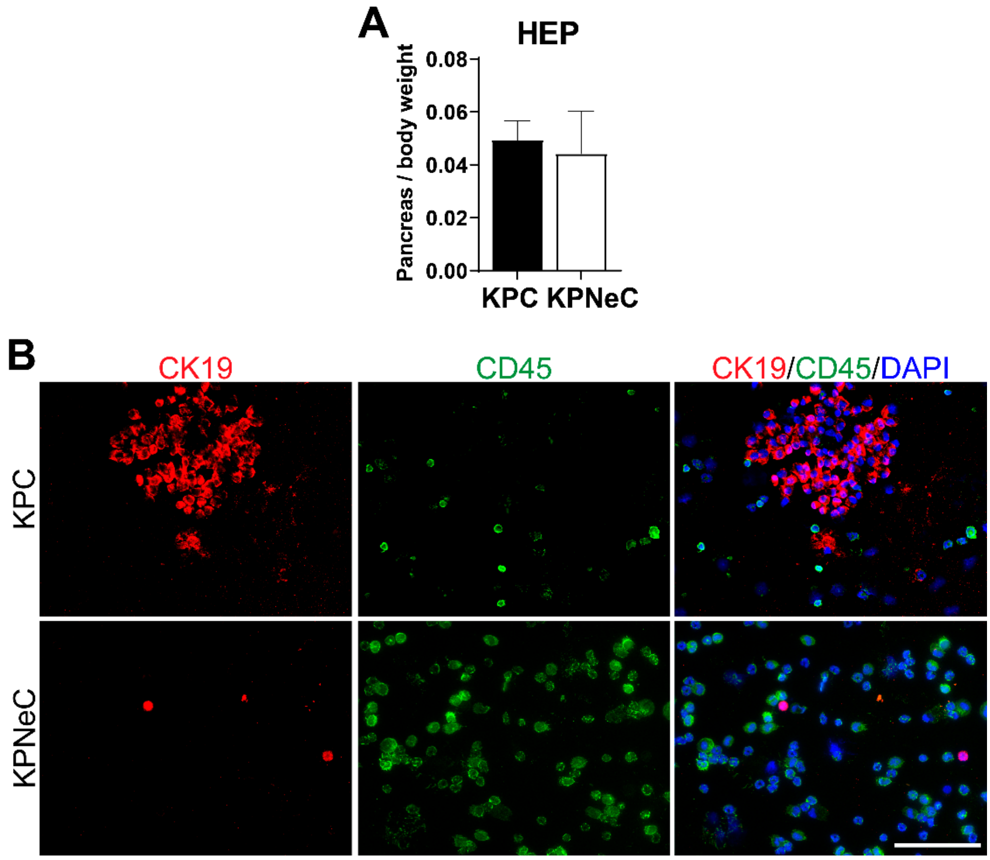

Figure S3. Analysis of KPC and KPNeC mice at their HEP. (A) Quantification of pancreatic weight to body weight ratio of KPC and KPNeC mice at their HEP. (N=9 mice/group). (B) Visualization of CK19⁺ and CD45⁺ ascitic cells isolated from HEP-analyzed KPC and KPNeC mice. Nuclear staining with DAPI, scale bar: 100 μm.

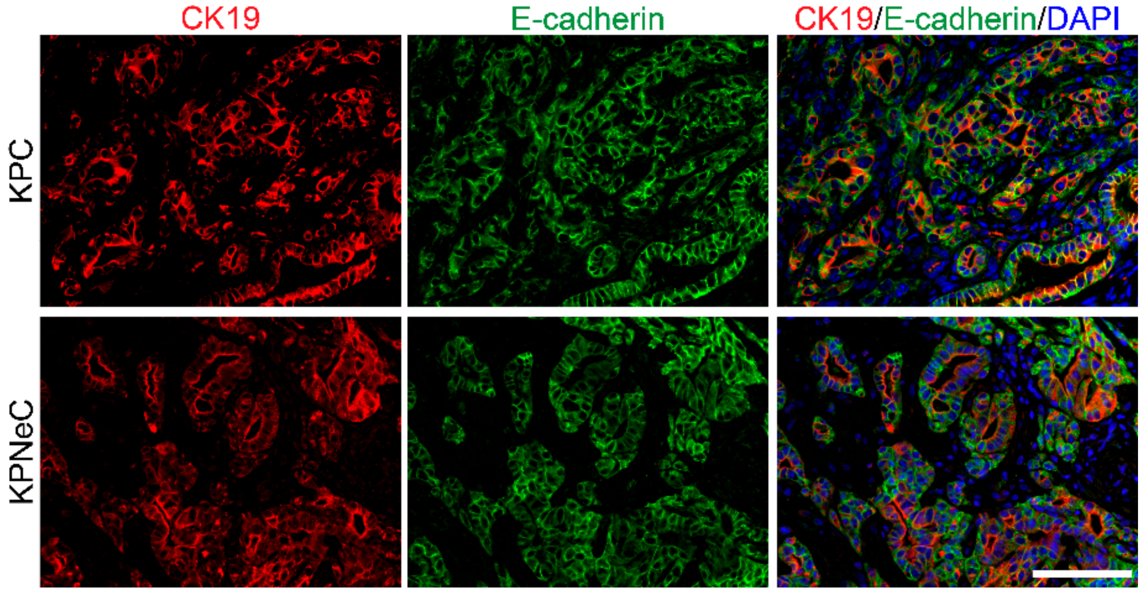

Figure S4. Visualization of CK19⁺ and E-cadherin⁺ cells on pancreatic sections of 12-week-old KPC and KPNeC mice. Nuclear staining with DAPI, scale bar: 100 μm.

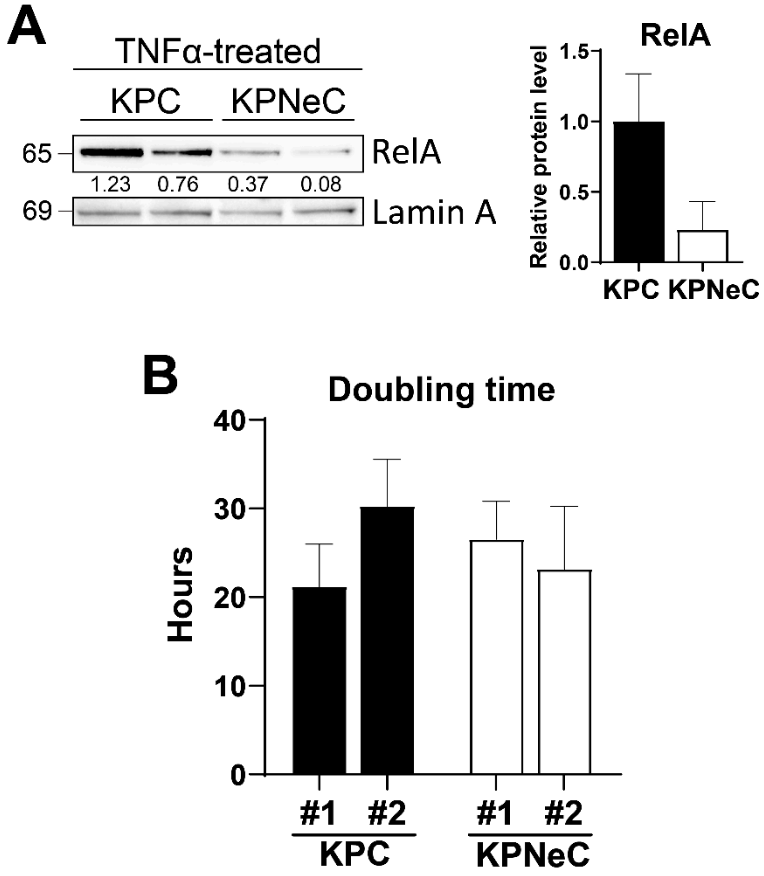

Figure S5. NEMO deletion inhibits NF-κB signaling in KPC primary cancer cells. (A) Left: Western blot analysis of nuclear protein extracts isolated from TNFα-treated KPC and KPNeC primary cancer cells. Lamin A was used as a loading control. Right: Quantification of the western blot analysis. The diagram shows the quantification of the RelA/Lamin A given relative to KPC cells, which were set to 1 (N=2/group). (B) Average time required for the doubling/proliferation of cancer cells derived from 2 different KPC and 2 different KPNeC mice.

Table S1. Primers and primary antibodies used in the study.

Gene	Forward primer sequence	Reverse primer sequence
Cdh1	agtgtttgctcggcgtct	gcaaagccatgaggagacc
Cdh2	aaccccatctcaggacagc	ccactgcatgtgctctcaag
Snai1	cttgtgtctgcacgacctgt	aggagaatggcttctcacca
Snai2	cattgccttgtgtctgcaag	cagtgagggcaagagaaagg
Timp1	gcaaagagctttctcaaagacc	agggatagataaacagggaaacact
Twist1	gctcagctacgccttctcc	tccttctctggaaacaatgaca
Vim	gtaccggagacaggtgcagt	ttctcttccatctcacgcatc
Zeb1	aggtgatccagccaaacg	ggtggcgtggagtcagag
Zeb2	gacagtcgcgtggaggag	gatgcagggggctgattat

Western Blot: Primary antibodies				
Antibody	Company	Catalog number	Dilution	Host Species
E-cadherin	Cell Signaling	3195S	1:1000	rabbit
GAPDH	Santa Cruz	sc-25778	1:1000	rabbit
Lamin A/C	Santa Cruz	sc-6215	1:1000	goat
NEMO/IKKγ	BD Biosciences	611306	1:1000	mouse
N-cadherin	Abcam	ab76011	1:1000	rabbit
RelA (p65)	Santa Cruz	sc-372	1:1000	rabbit
Slug	Cell Signaling	9585S	1:1000	rabbit

ZEB1	Santa Cruz	sc-25338	1:1000	rabbit

**Immunofluorescence: Primary antibodies**				
**Antibody**	**Company**	**Catalog number**	**Dilution**	**Host Species**
α-amylase	MilliporeSigma	3195S	1:400	rabbit
CD45	BD Biosciences	550539	1:100	rat
CK19	MilliporeSigma	sc-25778	1:150	rabbit
CK19	Santa Cruz	sc-33111	1:100	goat
Ki67	ThermoFischer	611306	1:100	mouse
Vimentin	Abcam	ab7783	1:100	rabbit

**Immunohistochemistry: Primary antibody**				
**Antibody**	**Company**	**Catalog number**	**Dilution**	**Host Species**
CD45	BD Biosciences	550539	1:100	rat
Snail+Slug	Abcam	ab85936	1:100	rabbit

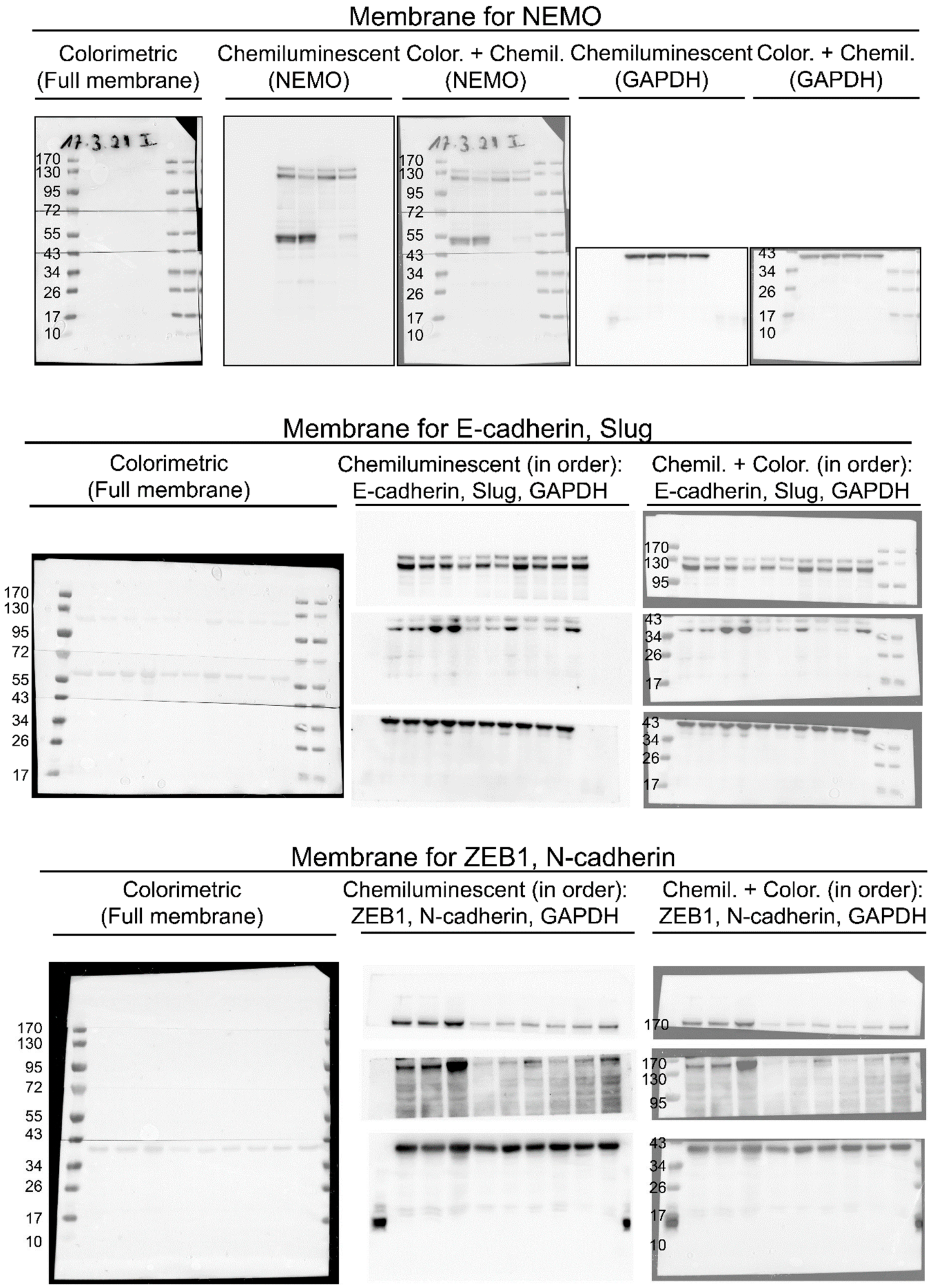

Membranes for western blot analysis depicted on Figure 4

Membrane for NEMO (pancreatic tissue)

NEMO

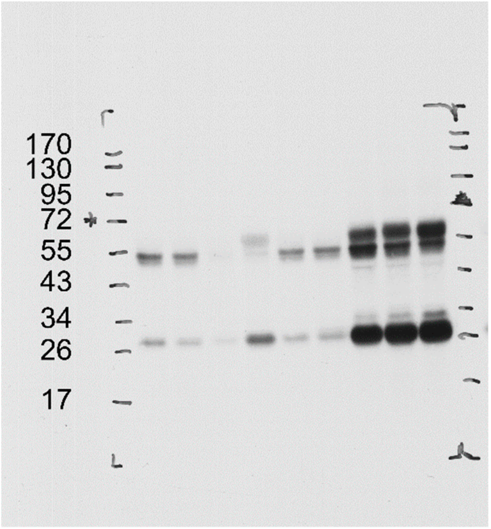

GAPDH

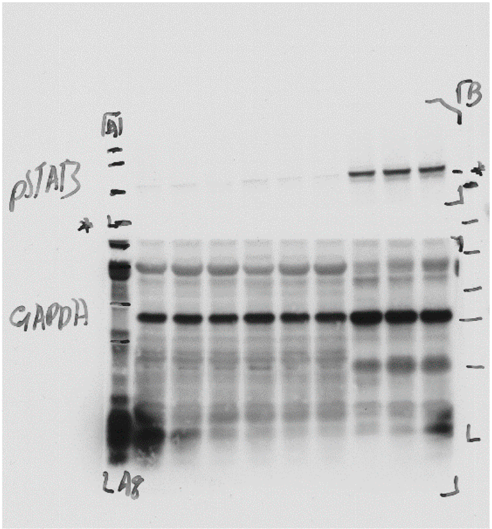

Membrane for western blot analysis depicted on Figure S2

Membrane for RelA

Colorimetric (Full membrane)

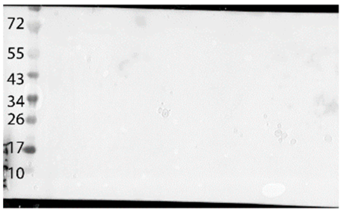

Chemiluminescent (RelA)

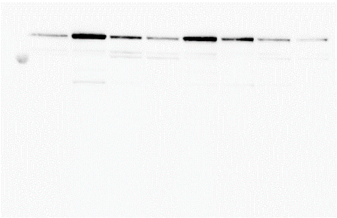

Chemilum. + Colorimetric (RelA)

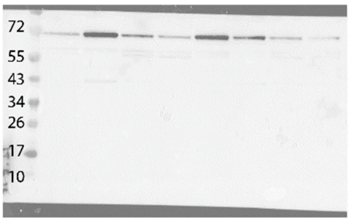

Chemiluminescent (Lamin A)

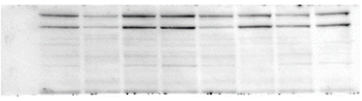

Chemilum. + Colorimetric (Lamin A)

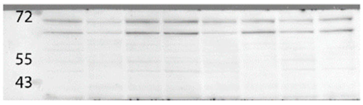

Membrane for western blot analysis depicted on Figure S5
